# Validation of a method for estimating peak skin dose from CT‐guided procedures

**DOI:** 10.1002/acm2.13261

**Published:** 2021-05-06

**Authors:** A. Kyle Jones, Meghan E. Kisiel, X. John Rong, Alda L. Tam

**Affiliations:** ^1^ Department of Imaging Physics The University of Texas MD Anderson Cancer Center Houston TX USA; ^2^ Department of Interventional Radiology The University of Texas MD Anderson Cancer Center Houston TX USA

**Keywords:** ablation, CT dosimetry, CTDI_vol_, CT‐guided intervention, peak skin dose

## Abstract

A method for estimating peak skin dose (PSD) from CTDI_vol_ has been published but not validated. The objective of this study was to validate this method during CT‐guided ablation procedures. Radiochromic film was calibrated and used to measure PSD. Sixty‐eight patients were enrolled in this study, and measured PSD were collected for 46 procedures. CTDI_vol_ stratified by axial and helical scanning was used to calculate an estimate of PSD using the method [1.2 × CTDI_vol_(helical) + 0.6 × CTDI_vol_(axial)], and both calculated PSD and total CTDI_vol_ were compared to measured PSD using paired t‐tests on the log‐transformed data and Bland‐Altman analysis. Calculated PSD were significantly different from measured PSD (*P* < 0.0001, bias, 18.3%, 95% limits of agreement, −63.0% to 26.4%). Measured PSD were not significantly different from total CTDI_vol_ (*P* = 0.27, bias, 3.97%, 95% limits of agreement, −51.6% to 43.7%). Considering that CTDI_vol_ is reported on the console of all CT scanners, is not stratified by axial and helical scanning modes, and is immediately available to the operator during CT‐guided interventional procedures, it may be reasonable to use the scanner‐reported CTDI_vol_ as an indicator of PSD during CT‐guided procedures. However, further validation is required for other models of CT scanner.

## 1 INTRODUCTION

The scope and complexity of computed tomography (CT)‐guided interventions is increasing,^1^ and CT is now used in combination with angiography in hybrid imaging systems.^2^, ^3^, ^4^, ^5^ Patient dose indices vary depending on the complexity of the procedure,^6^ and patients can be exposed to high peak skin doses (PSD) during the most complex procedures, as the same skin site is scanned repeatedly. This is very similar to the pattern of irradiation during dynamic imaging, including CT perfusion. The potential effects of high PSD delivered during such procedures were demonstrated in the CT perfusion incidents that occurred in the mid‐2000’s, with patients experiencing transient and permanent alopecia^7^ and an incident during which a young child experienced erythema after repeated CT of the head. Radiation cataractogenesis is a related concern if the area of repeated scanning includes the eye lenses.

Given the potential complications associated with high PSD experienced during CT, it is useful to understand how scanner‐reported dose indices relate to patient skin and surface doses, as these indices are available to the physician during the procedure, reported in real‐time. Soon after the CT perfusion incidents, studies examining patient doses resulting from CT brain perfusion were published, including a Monte Carlo study of patient lens and skin doses,^8^ a phantom study of organ doses,^9^ and a phantom study of skin dose.^10^ While these studies examined only CT brain perfusion imaging using axial scanning mode, an expression relating patient skin dose to the volume computed tomography dose index (CTDI_vol_) for interventional CT procedures was published around the same time,^11^ based on previous evaluation of the relationship between CTDI_vol_ and surface dose for different scanning modes.^12^ This method was based on a linear weighted sum of CTDI_vol_(helical) and CTDI_vol_(axial)^1^. The weighting factors were determined from the definitions of CTDI and CTDI_vol_, the relationship between peripheral and central CTDI measurements in the 32 cm body phantom, and the relationship between peripheral CTDI and surface dose reported by Bauhs et al.^12^ The method is as follows:

(1) $$\text{skin dose}=1.2\times {\text{CTDI}}_{\text{vol}}\left(\text{helical mode}\right)+0.6\times {\text{CTDI}}_{\text{vol}}\left(\text{intermittent}\left[\text{axial}\right]\text{mode}\right)$$

This method, while incorporated into existing recommendations regarding CT‐guided interventional procedures,^1^ has never been validated.

The purpose of this study was to validate this existing method for calculating patient PSD during CT‐guided interventional procedures. We hypothesized that the method would predict skin doses that were not significantly different from skin doses measured using radiochromic film.

## 2 METHODS

This study was conducted in compliance with the Healthcare Insurance Portability and Accountability Act and received prospective Institutional Review Board approval. Informed consent was obtained from all 68 patients enrolled in this study during a five month period. All procedures were performed on a single Definition AS128+ CT (Siemens Healthineers, Malvern, PA) located in the interventional radiology department. Procedures performed included radiofrequency, microwave, and cryo‐ ablation.

PSD were measured using Gafchromic XR‐RV3 film (Ashland, New Jersey). The film was cut into 35 cm × 5 cm strips. All film was from a single lot, and the film was calibrated using the same CT scanner used to perform the ablation procedures using standard methods.^13^ During calibration, strips of film were placed immediately beneath a 32 cm CTDI phantom, on top of the CT slicker and table pad, with the orange side facing down towards the table. Exposure was measured using a 0.6 cc thimble ionization chamber (10x5‐0.6, Radcal, Monrovia, CA) located adjacent to the film. The 0.6 cc chamber had a National Institute of Standards and Technology (NIST) traceable calibration at 120 kVp (HVL = 7.65 mm Al). Calibration films were exposed to air kerma ranging from 0 mGy to 4,009 mGy at 120 kV. The films were stored in a dark location for 2 weeks prior to scanning, as were films irradiated during patient procedures. All films were scanned using an Epson V700 Professional flatbed scanner (Epson, Long Beach, CA) in reflective mode in 48 bit color at 150 dpi with all corrections disabled. The long axis of the film was oriented parallel to the scan direction, which centered the narrow strips in the scanner. After scanning, film darkening was measured from the red channel data. A four parameter logistic model Rodbard fit^14^ was applied to the calibration data in ImageJ (NIH, Bethesda, MD)^15^ to derive the calibration function.

During CT‐guided interventions, one strip of film was placed in the same location and orientation as for calibration, immediately under the patient. The technologist was instructed to identify the lesion to be treated during the procedure on prior cross‐sectional imaging and then use anatomical landmarks to center the film, oriented along the long axis of the patient, as close as possible to the region of interest. As PSD was the dosimetric quantity of interest, it mattered only that the film was irradiated during a single rotation during each scanning event, i.e., capturing the entire length of long helical scans was not required. All images from every procedure were reviewed to ensure that the film was irradiated during each scan event. The area of maximum film darkening was identified visually and a region of interest containing at least 6,750 pixels was placed to measure the film darkening with the same methods used for calibration.

PSD was calculated using the method of Leng et al,^11^ listed previously in the introduction. This was accomplished by multiplying CTDI_vol_ from helical scans acquired during clinical procedures by 1.2 and adding this to the CTDI_vol_ from axial scans multiplied by 0.6 for all acquisitions during the procedure. The CDTI_vol_ reported for topograms was not included. CTDI_vol_ reported by the CT scanner used in this study was within 5% of the CTDI_vol_ measured during routine quality assurance testing performed during the study period. CT‐guided procedures at our institution generally follow the workflow outlined in the Best Practice Guidelines for CT‐Guided Interventional Procedures from the Society of Interventional Radiology,^1^ including, in order, a topogram, a helical preprocedure planning scan (PPS), axial or helical imaging during the intervention phase, and an optional postprocedure helical scan. Most procedures also included a limited multiphase contrast‐enhanced CT to assess ablation margin adequacy. Otherwise, most helical scanning was performed in i‐Spiral mode using a collimated beam width of 38.4 mm (detector configuration 32 x 1.2 mm) and a pitch of 1.0, and most axial scanning was performed using a collimated beam width of 14.4 mm (detector configuration 3 x 4.8 mm) in i‐Sequence mode. CT fluoroscopy was used occasionally, also at a beam width of 14.4 mm, and was treated the same as axial scanning. Most procedures used 120 kV, with occasional use of other kV depending on patient dimensions, and mAs was adapted to patient size using technique charts.^16^

Measured PSD were compared to calculated PSD and to total CTDI_vol_ alone using paired t‐tests on the log‐transformed data. A simple Bonferroni correction was used to correct for multiple comparisons. Five statistical tests were performed during data analysis, resulting in a corrected alpha of 0.05/5 = 0.01. Bland‐Altman analysis was used to examine bias, and correlation was calculated using the Spearman correlation coefficient.

## 3 RESULTS

**Fig. 1:**
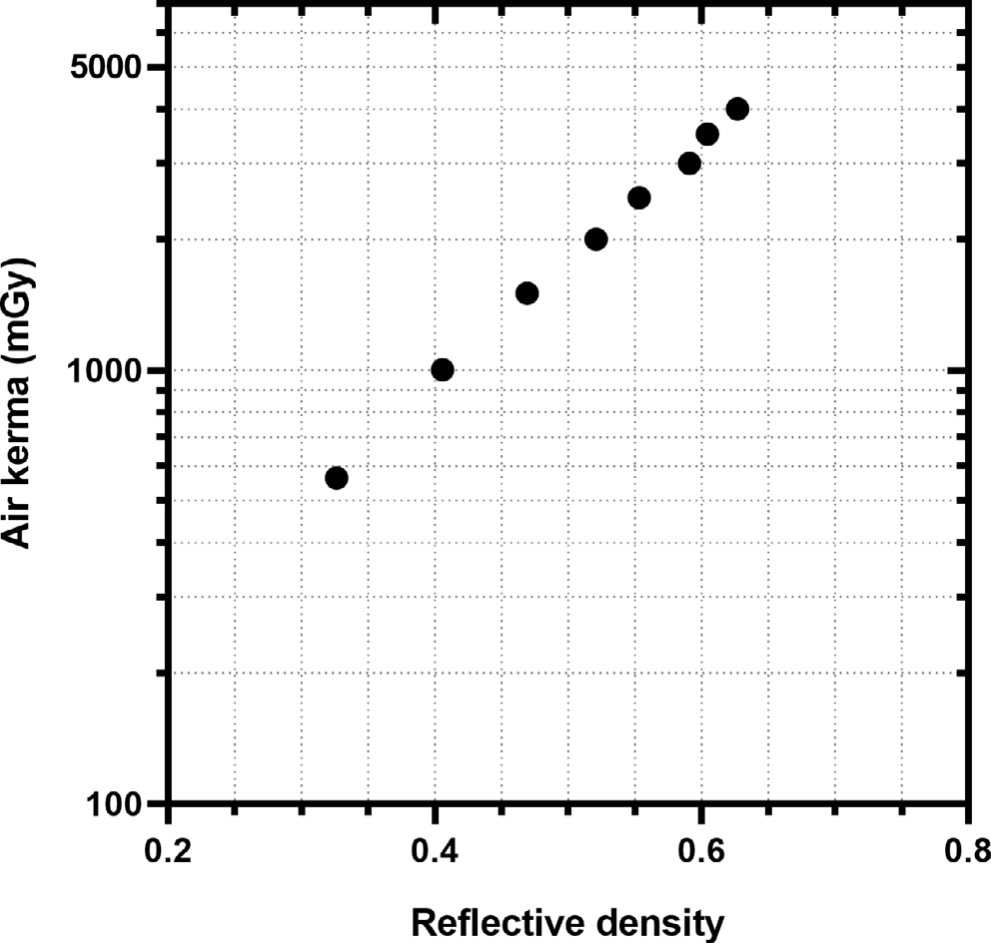
Radiochromic film calibration data plotted as air kerma versus reflective density.^13^

The film calibration data are plotted in Figure 1. One procedure was cancelled, the technologist forgot to place the film for 11 procedures, and no exposure was recorded on the film (i.e., the film was placed outside the imaged field of view) for 10 procedures, leaving 46 procedures available for analysis. Measured PSD ranged from 30.5 mGy to 1,303.9 mGy, with a median of 263.1 mGy.

**Fig. 2:**
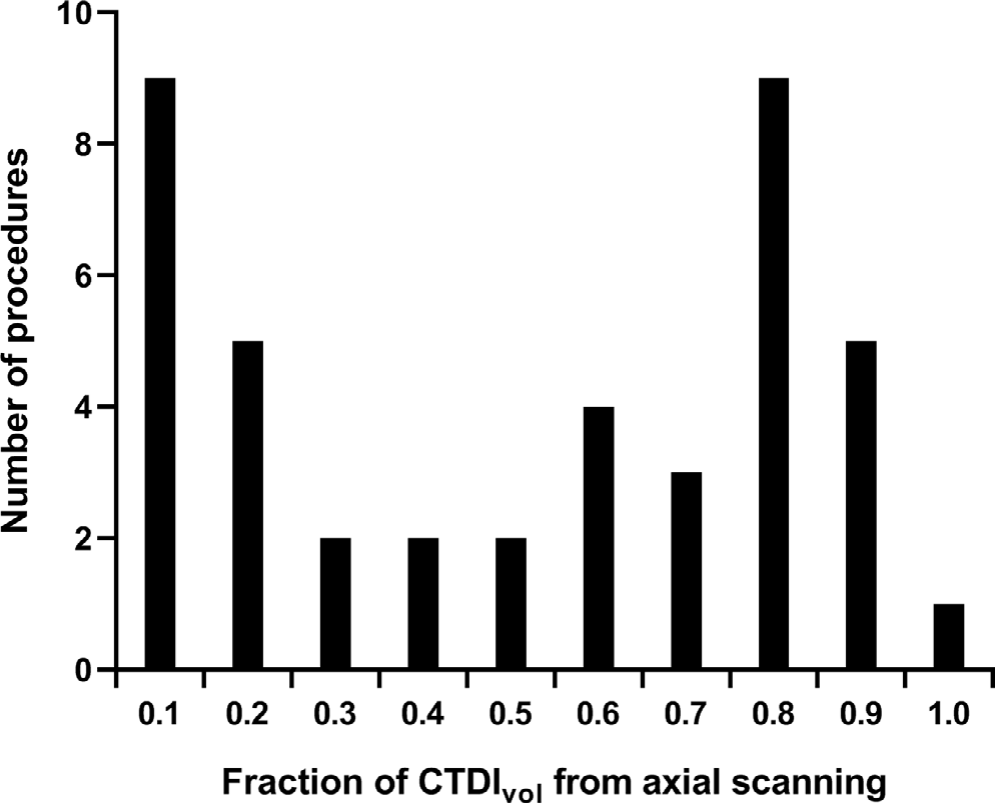
Histogram of the fraction of total procedural CTDI_vol_ contributed by axial scanning across all 46 procedures for which film dosimetry was available.

**Fig. 3:**
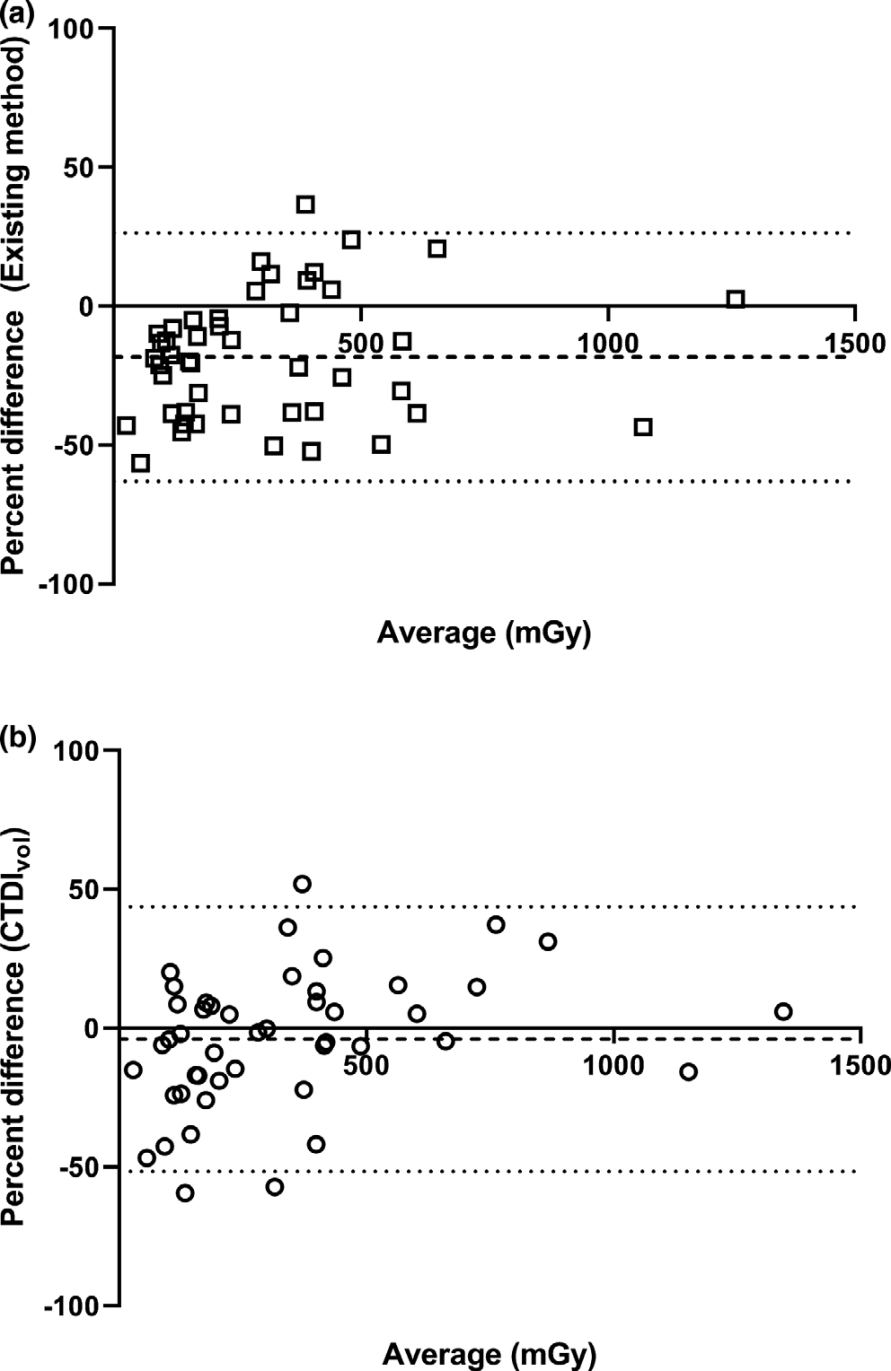
Bland‐Altman plots of (a) percent difference between peak skin dose (PSD) calculated using an existing method and measured PSD and (b) percent difference between total CTDI_vol_ and measured PSD. The dashed line indicates bias and the dotted lines indicate the 95% limits of agreement (LoA), and the x‐axis title “Average (mGy)” is the average of the estimates produced by the two measurement methods.

The fraction of CTDI_vol_ contributed by axial scanning varied widely (Fig. 2). Calculated PSD were significantly different from measured PSD (*P* < 0.0001). Calculated PSD underestimated the measured PSD by 18.3% (Fig. 3a, 95% limits of agreement, −63.0% to 26.4%). The standard deviation of the differences between calculated PSD and measured PSD across all procedures was 20.5 mGy. Total CTDI_vol_ was not significantly different from measured PSD (P = 0.27) and underestimated the measured PSD by 3.97% (Fig. 3b, 95% limits of agreement, −51.6% to 43.7%). The standard deviation of the differences between CTDI_vol_ and measured PSD across all procedures was 24.1 mGy.

## 4 DISCUSSION

In the specific context of this study, CTDI_vol_ alone was a better predictor of PSD during CT‐guided interventions than PSD calculated using an existing method. The 95% limits of agreement for the difference between CTDI_vol_ and measured PSD indicated that CTDI_vol_ can predict the PSD within 50%, a reasonable benchmark for patient dosimetry with indirect dose indices.^17^ While the existing method also, in general, predicted PSD within 50%, bias was greater, and use of the existing method required that CTDI_vol_ be stratified into contributions from axial and helical scanning, which is somewhat cumbersome and difficult to do during the procedure at the scanner console.

**Fig. 4:**
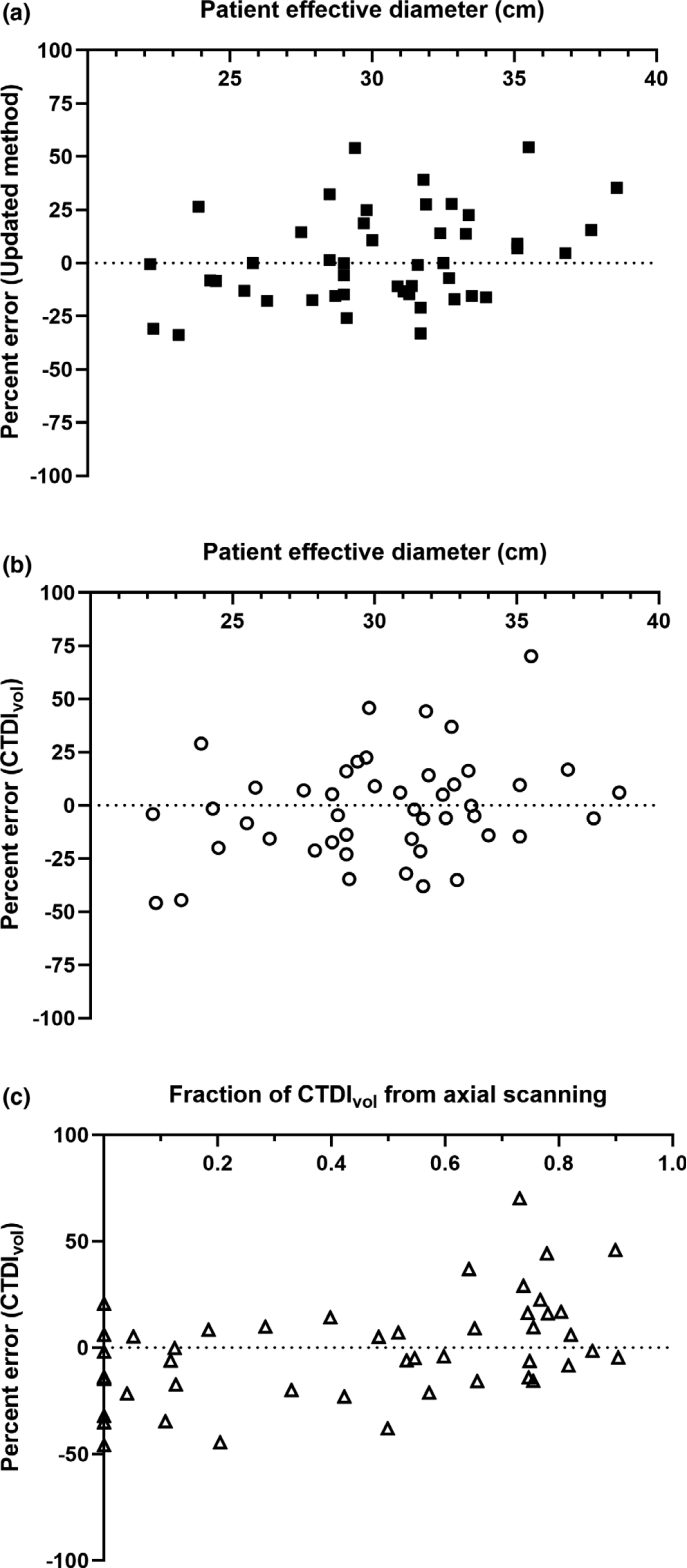
(a) Percent error in PSD was calculated using an existing method compared to measured PSD versus patient effective diameter; (b) percent error in CTDI_vol_ compared to measured PSD versus patient effective diameter; and (c) percent error in CTDI_vol_ compared to measured PSD versus fraction of CTDI_vol_ contributed by axial scanning.

It has been reported previously that surface dose as a percentage of CTDI_vol_ for axial scanning increases with decreasing phantom size, from 49% to 65% for phantom lateral widths ranging from 50 cm to 25 cm.^18^ There was a weak, non‐significant correlation ( ρ 0.30, P = 0.041) between percent error in calculated PSD compared to measured PSD and patient effective diameter (Fig. 4a). There was no correlation ( ρ 0.25, P = 0.093) between percent error in CTDI_vol_ compared to measured PSD and patient effective diameter (Fig. 4b). There was a weak, significant correlation ( ρ 0.44, P = 0.0023) between percent error in CTDI_vol_ compared to measured PSD and the fraction of CTDI_vol_ contributed by axial scanning (Fig. 4c). These findings likely reflect the complex interactions among the factors that affect the relationship between patient surface dose and CTDIvol, including scanning mode, patient size, and the position of the patient relative to isocenter, as patient access and probe placement considerations often mean that the patient is not centered in the gantry.

There are several uncertainties associated with radiochromic film dosimetry, these have been discussed extensively in a review by McCabe et al^13^ and summarized elsewhere.^19^ Considering that calibration of the film for the present study was done using backscatter conditions, the accuracy limits on measured PSD in this study were approximately ±10%, as calculated using the summary information in Table II of Ref. 18.

While this study included a variety of CT‐guided ablations performed on patients of different sizes, all procedures were performed using a single modern CT scanner at a single site, a limitation of this study. The relative contributions of primary and scatter radiation to CTDI_vol_ vary as a function of beam width ^20^, and it has been reported that surface dose as a percentage of CTDI_vol_ for axial scanning increases with increasing beam width, from 68% to 89% for beam widths ranging from 5 mm to 28.8 mm.^18^ However, the beam widths used for most scanning in this study, 14.4 mm for axial scanning and 38.4 mm for helical scanning, are very common in modern CT. Even so, our findings are still somewhat scanner‐specific, as other aspects such as beam quality, determined by X‐ray tube and generator design and bowtie filter shapes and materials, affect the relationship between peripheral and central CTDI_100_ and the relationship between surface dose and CTDI_vol_, as reported by las Heras et al.^10^ las Heras found that for the 16 cm CTDI head phantom the ratio of PSD to CTDI_vol_ varied by up to 25% among 4 CT scanner models. However, the scan protocols used in their study used 1, 2, or 4 gantry rotations to cover the imaged volume, leading to substantial variations in surface dose across the imaged volume.

## 5 CONCLUSION

Peak skin doses (PSD) resulting from CT‐guided procedures, calculated using an existing method, were significantly different from measured PSD for procedures performed using a single make and model of CT scanner. Total CTDI_vol_ was not significantly different from measured PSD in the specific context of this study.

## CONFLICT OF INTEREST

Alda L. Tam has the following disclosures: Research grant: BTG, Guerbet; Consulting: Boston Scientific, Endocare. None of the other authors has anything to disclose. This study was conducted in compliance with the Healthcare Insurance Portability and Accountability Act and received prospective Institutional Review Board approval.

## Data Availability

Data were available on request due to privacy/ethical restrictions.

## Note

^1^ See the penultimate paragraph of the Methods section for a brief description of how axial and helical CT are used together during CT‐guided procedures.

## References

[1] Jones AK , Dixon RG , Collins JD , et al. Best practice guidelines for CT‐guided interventional procedures. J Vasc Interv Radiol. 2018;29:518‐519. 29398413 10.1016/j.jvir.2017.10.021

[2] Inoue A . Development of a hybrid CT/angiography system. Med Rev. 1993;43:9‐15.

[3] Lin EY , Jones AK , Chintalapani G , Jeng ZS , Ensor J , Odisio BC . Comparative analysis of intra‐arterial cone‐beam versus conventional computed tomography during hepatic arteriography for transarterial chemoembolization planning. Cardiovasc Intervent Radiol. 2019;42:591‐600. 30413918 10.1007/s00270-018-2116-8

[4] Takayasu K , Muramatsu Y , Maeda T , et al. Targeted transarterial oily chemoembolization for small foci of hepatocellular carcinoma using a unified helical CT and angiography system: analysis of factors affecting local recurrence and survival rates. AJR Am J Roentgenol. 2001;176:681‐688. 11222205 10.2214/ajr.176.3.1760681

[5] Jones AK , Odisio BC . Comparison of radiation dose and image quality between flat panel computed tomography and multidetector computed tomography in a hybrid CT‐angiography suite. J Appl Clin Med Phy. 2020;21:121–127. 10.1002/acm2.12808 PMC7020994 31922349

[6] Yang K , Ganguli S , DeLorenzo MC , Zheng H , Li X , Liu B . Procedure‐specific CT dose and utilization factors for CT‐guided interventional procedures. Radiology. 2018;289:150‐157. 30015583 10.1148/radiol.2018172945

[7] Wintermark M , Lev MH . FDA investigates the safety of brain perfusion CT. AJNR Am J Neuroradiol. 2010;31:2‐3. 19892810 10.3174/ajnr.A1967 PMC7964089

[8] Zhang D , Cagnon CH , Villablanca JP , et al. Peak skin and eye lens radiation dose from brain perfusion CT based on Monte Carlo simulation. AJR Am J Roentgenol. 2012;198:412‐417. 22268186 10.2214/AJR.11.7230 PMC3918416

[9] Hoang JK , Wang C , Frush DP , et al. Estimation of radiation exposure for brain perfusion CT: standard protocol compared with deviations in protocol. AJR Am J Roentgenol. 2013;201:W730‐W734. 24063388 10.2214/AJR.12.10031

[10] de las Heras H , Minniti R , Wilson S , et al. Experimental estimates of peak skin dose and its relationship to the CT dose index using the CTDI head phantom. Radiat Prot Dosimetry. 2013;157:536‐542. 23864642 10.1093/rpd/nct171 PMC3853653

[11] Leng S , Christner JA , Carlson SK , et al. Radiation dose levels for interventional CT procedures. AJR Am J Roentgenol. 2011;197:W97‐W103. 21701002 10.2214/AJR.10.5057

[12] Bauhs JA , Vrieze TJ , Primak AN , Bruesewitz MR , McCollough CH . CT dosimetry: comparison of measurement techniques and devices. Radiographics. 2008;28:245‐253. 18203941 10.1148/rg.281075024

[13] McCabe BP , Speidel MA , Pike TL , Van Lysel MS . Calibration of GafChromic XR‐RV3 radiochromic film for skin dose measurement using standardized x‐ray spectra and a commercial flatbed scanner. Med Phys. 2011;38:1919‐1930. 21626925 10.1118/1.3560422 PMC3078021

[14] Rodbard D , Munson P , De Lean A . Improved curve‐fitting, parallelism testing, characterization of sensitivity and specificity, validation, and optimization for radioligand assay. In Radioimmunoassay and Related Procedures in Medicine. Vienna, Austria: International Atomic Energy Agency; 1978:469‐504.

[15] National Institutes of Health . ImageJ documentation. Available at https://imagej.nih.gov/ij/docs/index.html, accessed July 2020.

[16] Tam AL , Ensor JE , Zvavanjanja RC , et al. JOURNAL CLUB: standardizing CT‐guided biopsy procedures: patient dose and image noise. AJR Am J Roentgenol. 2015;205:W390‐W399. 26397346 10.2214/AJR.14.13324

[17] Balter S , Hopewell JW , Miller DL , Wagner LK , Zelefsky MJ . Fluoroscopically guided interventional procedures: a review of radiation effects on patients’ skin and hair. Radiology. 2010;254:326‐341. 20093507 10.1148/radiol.2542082312

[18] Leng S , Vrieze T , Yu L , McCollough C . Abstract SU‐GG‐I‐38: a direct skin dose calculation method in ct scans without table motion: influence of patient size and beam collimation. 2010 Annual Meeting of the American Association of Physicists in Medicine. Med Phys. 2010;37:3110.

[19] Jones AK , Ensor JE , Pasciak AS . How accurately can the peak skin dose in fluoroscopy be determined using indirect dose metrics? Med Phys. 2014;41:071913. 10.1118/1.4884020 PMC4105961 24989391

[20] Al‐Senan R . Abstract SU‐C‐12A‐01: primary vs. scatter contribution to body CTDI: experiment results. 2014 Annual Meeting of the American Association of Physicists in Medicine. Med Phys. 2014;41:106.

